# Effect of Endoscopic Gastroplication on the Genome-Wide Transcriptome in the Upper Gastrointestinal Tract

**DOI:** 10.1007/s11695-016-2356-0

**Published:** 2016-09-13

**Authors:** Nikkie van der Wielen, Givan Paulus, Mark van Avesaat, Ad Masclee, Jocelijn Meijerink, Nicole Bouvy

**Affiliations:** 1 Top Institute Food and Nutrition, Wageningen, The Netherlands; 2 Division of Human Nutrition, Wageningen University, Wageningen, The Netherlands; 3 Department of General Surgery, NUTRIM, Maastricht University Medical Center, Maastricht, The Netherlands; 4 Division of Gastroenterology and Hepatology, Department of Internal Medicine, NUTRIM, Maastricht, University Medical Center, Maastricht, The Netherlands

**Keywords:** Gastroplication, Transcriptome, Gene expression, Gastric tissue, Duodenum, HbA1c, Adiponectin, Immunity, Inflammation

## Abstract

**Background:**

Bariatric surgery is an effective intervention strategy in obesity, resulting in sustained weight loss and a reduction of comorbidities. Gastroplication, using the articulating circular endoscopic stapler, was recently introduced as a transoral bariatric technique. This procedure reduces gastric volume and induced 34.9 % of excess weight loss in the first year (Paulus et al. Gastrointest Endosc. 81(2):312–20, 3). The aim of the present study was to gain insight in the long-term effects and underlying mechanisms of gastroplication by investigating differences in the genome-wide gastric and duodenal transcriptome before and 1 year after intervention.

**Methods:**

Ten morbidly obese patients (BMI 39.8 ± 0.9 kg/m^2^ (mean ± SEM)) underwent gastroplication. Previous to the procedure and after 1 year, blood samples were taken, and mucosal biopsies were collected from the fundus, antrum and duodenum. Gene expression was measured using microarray analysis. Plasma adiponectin, HbA1c, IL-1β, IL-6, IL-7, TNF-α, IFN-γ, MCP-1, IL-8, TGF-1 and CRP levels were determined.

**Results:**

Downregulation of inflammatory genes and gene sets was observed in the fundus and duodenum 1 year after surgery. Gene expression of ghrelin and its activating enzyme GOAT were downregulated in the upper gastrointestinal tract. Patients showed a reduction in plasma HbA1c levels (from 6.17 ± 0.51 to 5.32 ± 0.14 %, *p* = 0.004) and an increase of plasma adiponectin (from 16.87 ± 3.67 to 27.67 ± 5.92 μg/ml, *p* = 0.002).

**Conclusions:**

Individuals undergoing gastroplication displayed a downregulation of inflammatory tone in the stomach and duodenum, which coincided with improved HbA1c and adiponectin levels. The reduction of inflammatory tone in the upper gastrointestinal tract may be a consequence of an improved metabolic health status or alternatively caused by the procedure itself.

**Electronic supplementary material:**

The online version of this article (doi:10.1007/s11695-016-2356-0) contains supplementary material, which is available to authorized users.

## Introduction

Bariatric surgery is the most effective medical option to achieve sustained weight loss in severe obesity. Besides traditional procedures such as laparoscopic Roux-en-Y gastric bypass (RYGB), vertical sleeve gastrectomy (VSG) and adjustable gastric banding (LAGB), less invasive options are available such as the (transorally placed) duodenal-jejunal bypass sleeve (DJBS). In general, these procedures lead to a loss of body fat, a reduction of comorbidities and improvement of long-term health risks. Remarkably, the mechanisms behind these outcomes are still poorly understood, and it is conceivable that these comprise different combinations of biological adaptations [1]. This is reflected in the markedly different immediate effects on glycaemic control following different procedures [2]. Recently, endoscopic gastroplication has become available as a new minimal invasive technique. The articulating circular endoscopic (ACE) stapler is used to reduce the volume of the stomach without removing tissue or bypassing other intestinal regions. For this procedure, no skin incisions are necessary; it is performed via a transoral route. This procedure results in a median 34.9 % (IQR 17.8–46.6) loss of excess weight in the first year. Moreover, only mild adverse effects were reported so far [3]. Although several studies have described metabolic and anti-inflammatory effects of bariatric surgery at a molecular level, studies on these processes within the gastrointestinal (GI) tract are still limited. This holds particularly true for the upper GI tract, as most studies in this field have focussed on the mid or lower gastrointestinal tract [4–6]. Moreover, these studies concern effects of RYGB, a procedure extensively changing GI anatomy and physiology. The present study was undertaken to gain more insight in the long-term effects and underlying mechanisms of gastroplication in the upper GI tract: the stomach (fundus and antrum) and the duodenum, and to relate these to general health outcomes, including parameters of inflammation. To this end, transcriptome and gene set enrichment analysis was performed with biopsies obtained before and 1 year following gastroplication.

## Materials and Methods

### ACE Stapler Study

This study used biopsies and blood samples obtained from ten patients who were part of the first human ACE stapler study [3]. The Medical Ethical Committee of the Maastricht University Medical Center+ in the Netherlands (NCT02381340) approved the present study as a sub-study aiming to further unravel underlying mechanisms. Before inclusion, written informed consent was obtained from each participant. The inclusion criteria for the ACE stapler study are described in detail by Paulus et al. [3]. In brief, participants were 18 to 50 years old with a BMI of 40 to 45 kg/m^2^ or 30 to 39.9 kg/m^2^ in combination with one or more comorbidities expected to improve with weight loss. The ACE stapler was introduced into the stomach together with a thin endoscope. By applying vacuum to the gastric tissue, a large full-thickness (transmural) plication was drawn into the stapler head and fixed with a staple ring. Reduction of the stomach volume along the greater curvature was completed after creating a maximum of eight plications in the fundus and two additional plications in the antrum of the stomach. More details on the procedure were published previously [3]. Mucosal biopsies were taken from the fundus, antrum and duodenum with a standard forceps before starting the procedure. Afterwards, patients visited the outpatient clinic regularly and were stimulated to adhere to a healthy lifestyle. A follow-up endoscopy was planned 12 months after the procedure, at which the biopsy procedure was repeated. Biopsies were snap frozen in liquid nitrogen and stored at −80 °C until analysis. Table 1 shows a brief overview of the characteristics of included patients.

**Table 1:** Overview of characteristics of patients undergoing ACE stapler procedure. Measurements were performed at baseline and 1 year after the procedure. Ghrelin was measured after a 10 hour overnight fast

	Baseline		One year	
	Mean	SEM	Mean	SEM
Age	39	2		
Male/female ratio	6:4			
BMI (kg/m^2^)	39.8	0.9	33.4	0.9
Excess weight loss (%)			37.9	4.8
Fasted active ghrelin level (pg/ml)	46.5	5.9	63.4	5.2

### RNA Isolation and Microarray Processing

RNA of the mucosal biopsies was isolated using TRIzol reagent (Life technologies, Bleiswijk, Netherlands) and further purified using the RNeasy micro kit (Qiagen, Venlo, Netherlands). RNA yield was measured with the Nanodrop ND-1000 Spectrophotometer, and the quality of the RNA samples was verified with an Agilent 2100 Bio analyser (Agilent Technologies, Amstelveen, Netherlands). One hundred nanogram of RNA was used for whole transcript cDNA synthesis (Affymetrix, Inc., Santa Clara, USA). Hybridization, washing and scanning of Affymetrix GeneChip Human Gene 1.1 ST arrays was carried out according to standard Affymetrix protocols.

### Microarray Analysis

For the analysis of the microarray results, each location (i.e. fundus, antrum and duodenum) was analysed separately. Arrays were normalized using the robust multi-array average method [7, 8]. Probe sets were assigned to unique gene identifiers, in this case Entrez IDs. The probes on the arrays represent 19654 Entrez IDs [9]. Array data were analysed using MADMAX pipeline for statistical analysis of microarray data [10]. Quality control was performed, and all arrays met our criteria, except for the fundus and antrum arrays from participant 5, which were excluded. All data were filtered, and probe sets with expression values above 20 in at least 5 arrays were included for further analysis. These data were used for gene set enrichment analysis (GSEA; www.broadinstitute.org/gsea [11]) in MADMAX. Gene sets with a false discovery rate (FDR) <0.25 were considered significantly enriched. The gene set enrichment analysis was visualized using the enrichment plugin in Cytoscape with conservative filtering (*p* < 0.001 and FDR q < 0.05). For further analysis of individual genes, a cut-off of IQR > 0.25 was used to filter out genes that showed no variation between the samples; Intensity-Based Moderated T-statistics (IBMT) was used to assess significant differences with *p* value <0.05.

### Plasma Measurements

Blood samples were collected in EDTA-coated tubes, centrifuged and stored at −80 C until analysis. The measurement of plasma adiponectin and cytokine levels was performed using an in-house developed and validated multiplex immunoassay (Laboratory of Translational Immunology, University Medical Center Utrecht, the Netherlands) based on Luminex technology (xMAP, Luminex, Austin, USA). The assay was performed as described previously [12]. Using heteroblock (Omega Biologicals, Bozeman, USA), aspecific heterophilic immunoglobulins were preabsorbed. Acquisition was performed with the Biorad FlexMAP3D (Biorad laboratories, Hercules, USA) in combination with xPONENT software version 4.2 (Luminex, Austin, USA). Data was analysed by 5-parametric curve fitting using Bio-Plex Manager software, version 6.1.1 (Biorad laboratories, Hercules, USA). HbA1c levels were determined routinely at the Department of Clinical Chemistry of the Maastricht University Medical Center. Active ghrelin was measured using an established in-house radioimmunoassay (Millipore, Massachusetts, USA).

### Statistical Analysis

Statistical analyses were performed using Prism 5.0 (GraphPad Software, Inc. La Jolla, USA). The effects of treatment on plasma levels of inflammatory markers and adiponectin were tested by paired *t* tests for normally distributed variables and Wilcoxon’s signed rank tests for non-normally distributed variables. Spearman’s rank correlation coefficient analysis was performed to investigate the association between changes in biochemical parameters with changes in mRNA expression (signal log ratio). A *p* value <0.05 was considered statistically significant. Data are presented as mean with standard error of the mean (SEM). Statistical analysis of transcriptome data was described above.

## Results

### Effects of Gastroplication on Systemic Metabolic and Inflammatory Parameters One Year After Intervention

Plasma levels of glycated haemoglobin, adiponectin and several pro-inflammatory mediators before and after intervention are shown in Table 2. Significant changes were found for adiponectin and HbA1c. Adiponectin showed a 1.64-fold increase (*p* = 0.002) in the patients who underwent ACE stapler treatment. Glycated haemoglobin (HbA1c) was significantly decreased (*p* = 0.004) by the treatment. Plasma IL-6 showed a tendency to decrease following ACE stapler treatment by a factor 1.47. MCP-1 levels also showed a decrease (1.3-fold), but this effect did not reach statistical significance.

**Table 2:** The effect of ACE stapler treatment on fasted plasma levels of inflammatory and metabolic markers. Plasma levels were measured before the treatment (baseline) and 1 year after

Plasma marker		Baseline		One year		Treatment effect	
	Unit	Mean	SEM	Mean	SEM	Difference	*p* value
Adiponectin	μg/ml	16.87	3.67	27.67	5.92	10.80	0.002*
HbA1c	%	6.17	0.51	5.32	0.14	−0.85	0.004*
IL-1β	pg/ml	1.42	0.06	1.37	0.08	−0.05	0.244
IL-6	pg/ml	10.90	1.83	7.41	1.80	−3.49	0.069
IL-7	pg/ml	11.52	1.24	10.43	2.01	−1.09	0.180
TNF-α	pg/ml	2.14	0.10	2.00	0.14	−0.14	0.118
IFN-γ	pg/ml	2.54	0.27	2.23	0.25	−0.32	0.099
MCP-1	pg/ml	60.70	7.33	46.68	7.74	−14.02	0.088
IL-8	pg/ml	7.03	1.03	6.55	1.44	−0.48	0.455
LAP/TGF-1	ng/ml	3.11	0.35	3.24	0.36	0.13	0.393
CRP	mg/l	12.62	5.66	8.78	2.75	−3.85	0.248

### Effects on Tissue Gene Expression Mainly Relate to Inflammatory Pathways

**Fig. 1:**
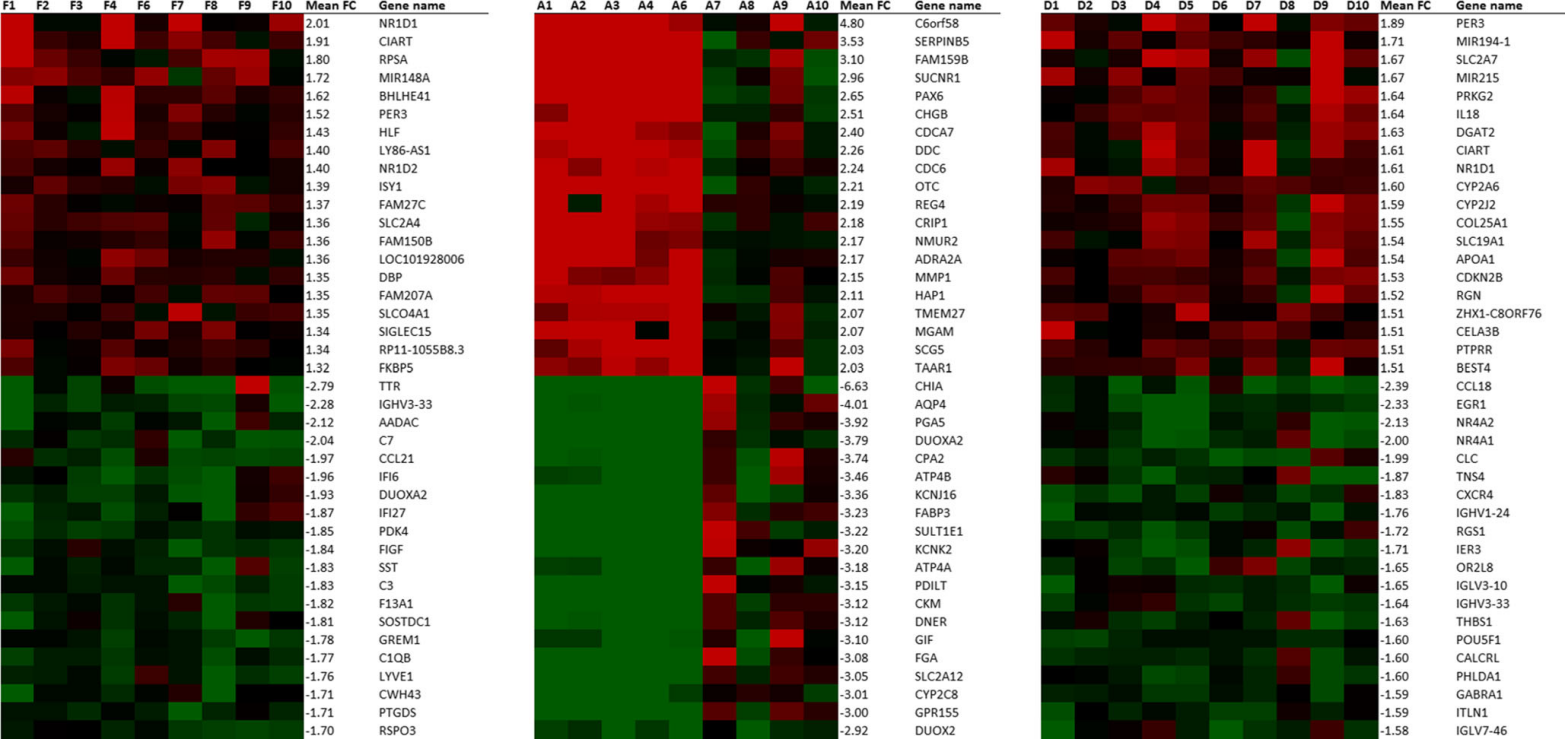
Expression of most highly significantly upregulated and downregulated genes in fundus, antrum and duodenum, respectively. *Green* is a signal log ratio of −2, and *red* is a signal log ratio of 2. Apart from the mean fold change (FC) of the top regulated genes by the treatment, signal log ratios are displayed to show inter-individual differences

Gene expression changes 1 year after intervention compared to baseline were analysed for different locations of the upper gastrointestinal tract, namely fundus, antrum and duodenum. After intervention, 727 genes (259 upregulated, 468 downregulated) were significantly changed in the fundus, 1846 (951 upregulated, 895 downregulated) in the antrum and 921 genes (480 upregulated, 441 downregulated) in the duodenum. The top 20 upregulated and downregulated genes in all three locations are shown in Fig. 1. In both fundus and duodenum, a considerable number of downregulated genes have been associated with immunity and inflammatory pathways. In the fundus, the expression of immune-related genes like *IGHV3-33*, *C7*, *CCL21*, *IFI6*, *IFI27*, *C1QB* was downregulated, and *CCL18*, *CLC*, *CXCR4*, *IGHV1-24*, *RSG1*, *IGLV3-10*, *IGHV3-33*, *IGLV7-46* were downregulated in the duodenum. In the antrum, there was an upregulation of some neuroendocrine-associated genes, namely *PAX6*, *CHGB*, *SCG5*.

### Gene Set Enrichment Analysis Reveals Potential Processes Involved

**Fig. 2:**
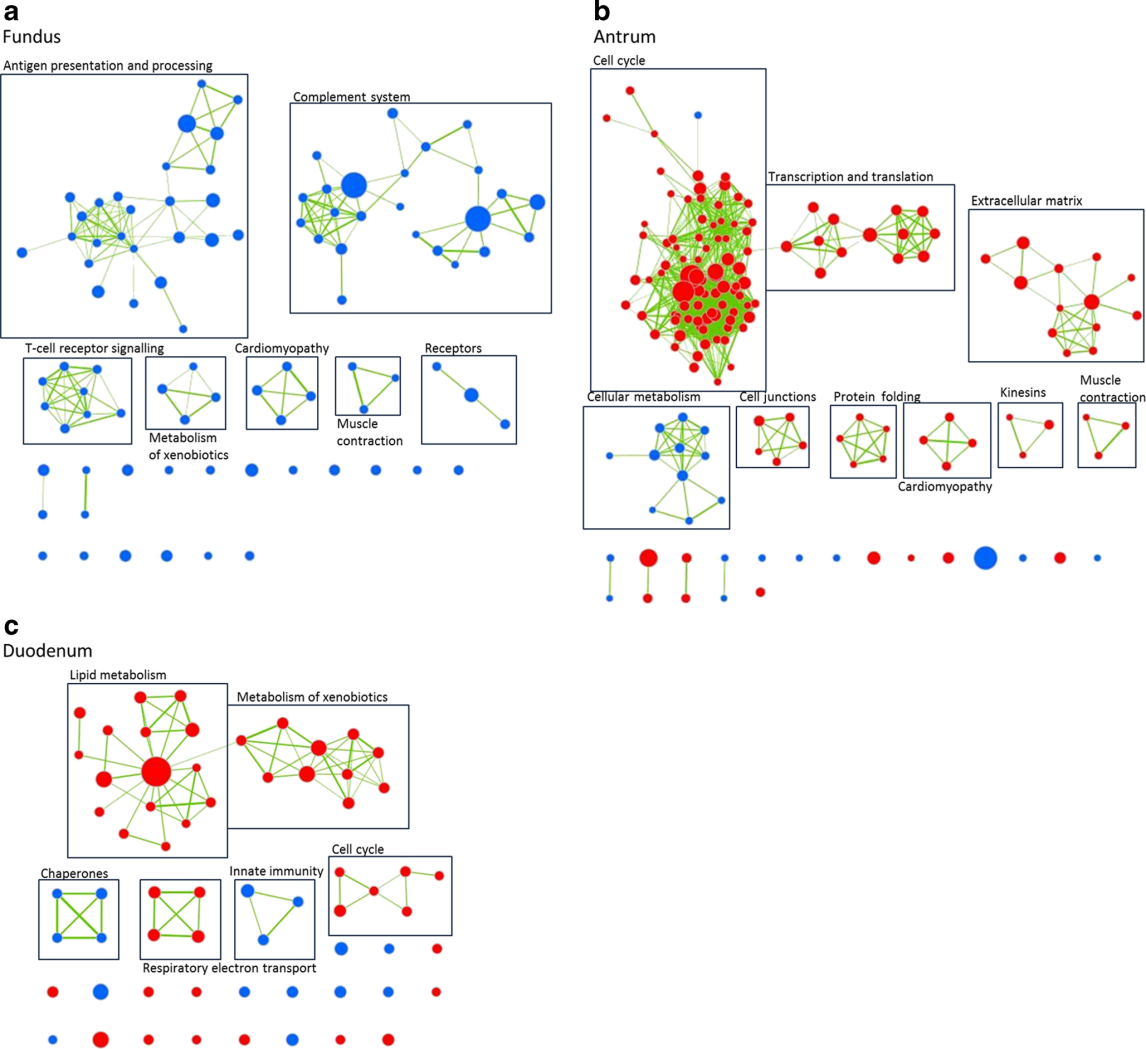
Gene set enrichment analysis of the fundus, antrum and duodenum. Each node indicates a conservative filtered gene set (*p* < 0.001 and FDR q < 0.05), and the *connecting lines* indicate overlapping genes between the nodes/gene sets. *Red* is enriched; *blue* is depleted gene sets

To gain more insight into the processes changed 1 year following the stomach volume reduction procedure, gene set enrichment analysis (GSEA) was performed. This computational method uses molecular signatures to associate changes in gene expression with known biological processes. Analysis resulted in 236 (2 upregulated, 234 downregulated) enriched gene sets for fundus, 546 (474 upregulated, 72 downregulated) enriched gene sets for antrum and 253 (182 upregulated, 71 downregulated) enriched gene sets for duodenum. In the antrum, more gene sets were upregulated, whereas in the fundus, most gene sets were downregulated (Fig. 2). Of these downregulated gene sets in the fundus, many were related to immune responses, mostly to the complement system, presentation and recognition of antigens (self or pathogenic) and T cell receptor signalling. Also in the duodenum, some of the downregulated gene sets were related to the innate immunity. In the antrum, cell cycle related gene sets were strongly enriched. In the duodenum, the enrichment analysis showed also a slight upregulation of cell cycle processes. Here, more metabolic pathways were apparently upregulated, including those associated with ‘fat digestion and absorption’ and ‘metabolism of lipids and lipoproteins’. All gene sets are specified in Table S1.

### Changes in Gastrointestinal Hormone Expression

**Fig. 3:**
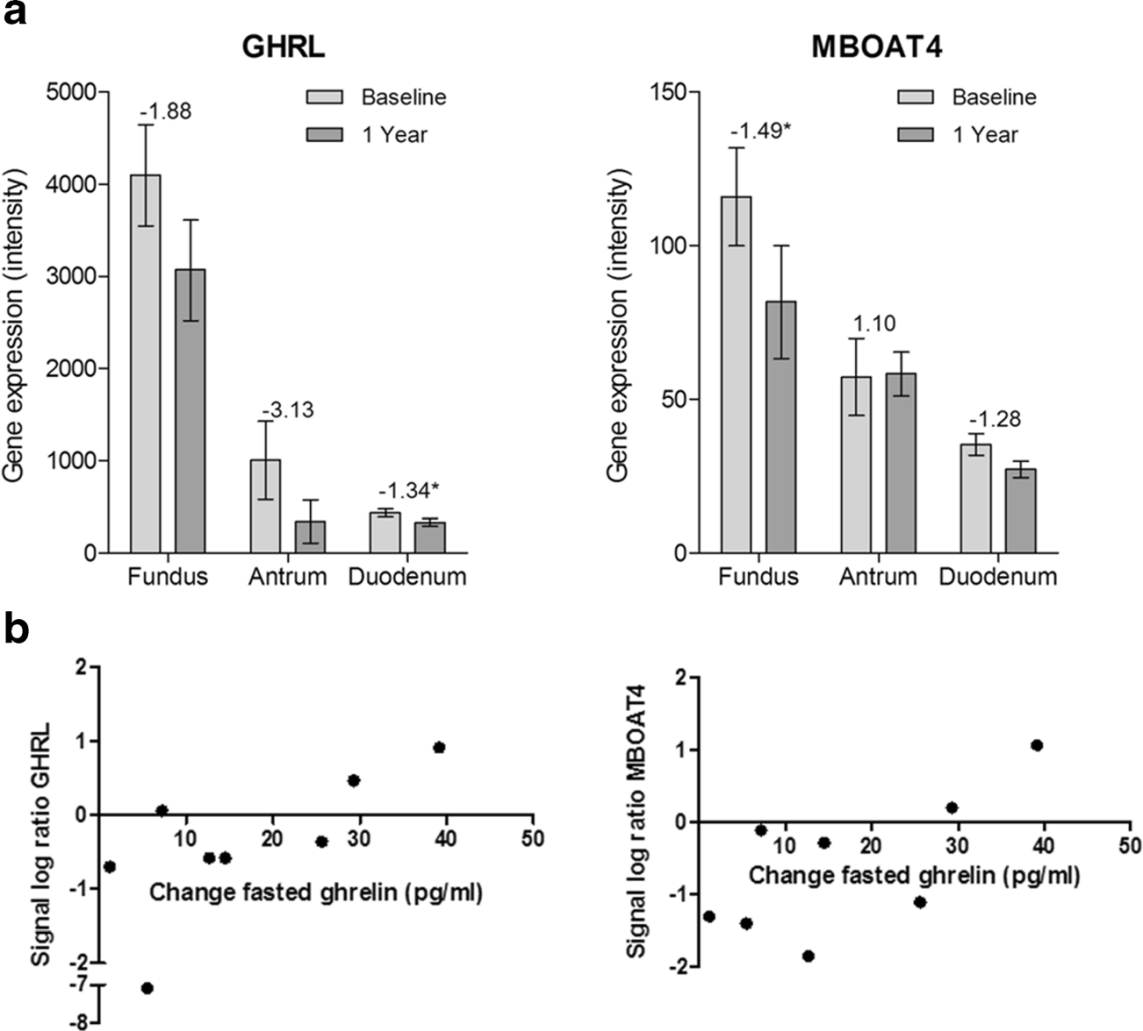
Changes in gene expression of ghrelin and MBOAT4 and their correlation to plasma ghrelin. **a** The graphs show RMA normalized intensities of microarray data at baseline and 1 year after gastroplication in biopsies of fundus, antrum and duodenum. Fold changes are indicated on *top of the bars*, significant changes are marked with *asterisk* (*p* < 0.05). All graphs show mean and SEM. **b** Correlation between signal log ratios of ghrelin (GHRL) and MBOAT4 in the fundus with changes in plasma active ghrelin levels measured in a fasted state before and 1 year after the procedure

Being one of the main gastric hormones, ghrelin is not only involved in appetite regulation but also in immunity [13]. Two genes related to ghrelin were significantly changed in specific locations of the GI tract (Fig. 3a). In the fundus, there was a downregulation of *MBOAT4* (FC = −1.49), the gene encoding the ghrelin-activating enzyme GOAT4. Furthermore, there was a trend for downregulation of ghrelin (*GHRL*) expression itself in the fundus (FC = −1.88, *p* = 0.19) and antrum (FC = −3.13, *p* = 0.11) and a significant downregulation in the duodenum (FC = −1.34).

In the fundus, the ghrelin gene expression (*GHRL*) was positively correlated to fasted plasma changes of active ghrelin (Spearman correlation coefficient = 0.826, *p* = 0.015), and for MBOAT4 expression, there was a tendency for correlation (Spearman correlation coefficient = 0.69, *p* = 0.069) (Fig. 3b). Although the correlation between changes in ghrelin and GHRL expression seems to be influenced by a single patient, excluding this patient still resulted in a significant correlation.

## Conclusions

Results of the present study add important physiological background information to the clinical outcomes observed in patients after undergoing ACE stapler gastroplication. Our plasma analyses revealed beneficial effects on HbA1c and adiponectin levels 1 year after surgery, indicating an improvement of glycaemic control and a favourable shift in adipose tissue mass and/or inflammatory status, respectively. This rise in plasma adiponectin and reduction of HbA1c is in line with previous studies in which a loss of excess body fat was achieved, including those involving other surgical and non-surgical weight loss interventions [14–17]. We also found tendencies for reduced plasma IL-6 and MCP-1 levels, 1 year after intervention. Reduced levels of inflammatory markers are generally assumed to result from a reduction of visceral fat mass in particular, which plays a major role in the low grade inflammatory state associated with obesity [18]. The total number of patients in our study population is quite small, and it is conceivable that statistical significance might have been reached with a larger patient group. Several other studies have found significant reductions in inflammatory markers like CRP, MCP-1 and IL-6 after bariatric surgery, while others did not find such an effect [19–21].

To our knowledge, this study is the first to analyse long-term whole transcriptome changes in the upper gastrointestinal tract after a new transoral bariatric procedure. Gastroplication reduces gastric volume without altering intestinal anatomy, as is the case with RYGB. In contrast to bypass surgery, exposure of the intestinal epithelium to nutrients and their metabolites is largely maintained after this endoscopic gastroplication. At the same time, small changes related to different GI transit characteristics or changes in the microbiome might still occur.

Analysis of the large amount of data using unbiased transcriptome analysis clearly pointed towards a reduction in inflammatory tone in the fundus and duodenum tissues as manifested by the downregulation of a wide variety of inflammation-related gene sets. The downregulated gene sets in the fundus were mostly related to innate immunity, and particularly associated with downregulation of the complement system, presentation and recognition of antigens (self or pathogenic), IFN-γ signalling and T cell receptor signalling. In the duodenum, the main downregulated gene set was associated with the complement system. Moreover, the top 20 highly changed genes in these locations also suggest notable downregulation of many immune-related processes, of which several were related to chemokines, complement system, interferon signalling and immunoglobulins. These results coincided with the weight loss, improvement of HbA1c levels and decrease of whole-body inflammatory tone in these patients.

Based on the present study, we cannot establish whether the apparent reduction of inflammatory tone in the upper GI tract has a predominantly local cause, i.e. due to a changed food-intake pattern or digestion process, or whether it is related to a reduction of low-grade systemic inflammation due to the reduction of body fat mass.

Increasing evidence points to a link between intestinal inflammation status in general, obesity and (or) diabetes. In obesity, increased innate cell densities, among which macrophages, natural killer cells and T cells, especially the proportion of cytotoxic CD8 T cells, have been observed in the jejunal epithelium. These epithelial T cells were found to be associated with local and systemic comorbidities. Furthermore, isolated T cells from obese patients decreased insulin sensitivity of epithelial cells in vitro [22]. Another study reported that diet-induced weight loss resulted in a downregulation of inflammatory pathways and inflammatory cytokines IL-8, TNF-α, MCP-1 and IL-1β, in recto-sigmoid mucosal tissue [23]. Moreover, increased intestinal inflammatory gene expression of TNF-α, IL-6, ICAM and PTGS-2 was found in insulin-resistant obese patients compared to non-insulin-resistant obese patients, suggesting that intestinal inflammation is involved in diabetes during obesity [24, 25]. A prominent feature of the immune system in the gastrointestinal tract is to provide adequate protection without stimulating excessive inflammation, thereby maintaining a fine balance [26, 27]. A pro-inflammatory immune status of the gastrointestinal tract in obese patients might be protective against increased luminal challenges associated within obesity but deteriorating for insulin resistance [22]. Furthermore, this pro-inflammatory status might be linked to the increased prevalence of inflammatory bowel disease and cancer in obese patients [28–30]. In summary, we can only speculate whether the reduced inflammatory microenvironment in gastric and duodenal tissue found after gastroplication can be considered as a positive or negative outcome.

In the antral tissue, gene sets related to cell cycle processes and extracellular matrix were increased. This might be explained by dilation of the stomach, a common observation after gastric volume reduction [31–34]. While gastric volume was not quantified, we perceived the stomach as larger at 1-year follow-up than immediately after gastroplication.

An interesting observation was that the mean gene expression of ghrelin and the enzyme GOAT, responsible for ghrelin acylation, decreased after gastroplication in some of the tissues. At the same time, plasma fasted active ghrelin was increased, and there was a positive correlation between the gene expression of ghrelin and plasma values of active ghrelin. Ghrelin is one of the most prominent hormones secreted from the upper gastrointestinal tract and does not only play a role in appetite regulation but also in inflammation [13]. Consistent with our results, in RYGB patients, significant lower levels of jejunal ghrelin gene expression have been reported after 10 months [6]. Furthermore, GOAT mRNA expression and GOAT-positive cell numbers were lower in a non-obese group compared to morbidly obese patients, although no changes in jejunal ghrelin expression were detected [35]. Moreover, more ghrelin positive cells were found in the stomach of morbidly obese and overweight patients compared to healthy normal weight controls [36, 37], which might indicate that with weight loss, the number of ghrelin-releasing cells will decrease. The discrepancy with ghrelin expression in the gastrointestinal tract and plasma ghrelin values might be explained by a reduced secretory activity of (a higher number of) ghrelin producing cells in obesity, as suggested by Widmayer et al. [36]. However, within our patients, there was a positive correlation between its gene expression and plasma levels, indicating that upregulated expression of ghrelin in the fundus was associated with greater increase in fasted ghrelin levels and downregulated expression with a smaller increase 1 year after gastroplication. The underlying cause of the observed changes cannot be pinpointed in our study. It is possible that the changes in ghrelin are dependent on the surgical procedure, which takes place at the main site of ghrelin secretion. Furthermore, the implications of these changes in ghrelin are not fully understood and need further investigation to crystallize the underlying mechanism and to explore the potential of these changes in obesity treatment.

There are some strengths and limitations to this study. The within-person measurement of changes in gastrointestinal gene expression is unique as most studies in this field are observational. By applying a prospective design, we were able to perform paired analysis and look specifically for changes induced by the gastroplication treatment instead of comparing obese subjects with lean controls. Whole transcriptome analysis enabled us to investigate changes in an unbiased manner. One of the limitations of this study is that it was not powered to find differences in inflammatory markers. Therefore, the inclusion of more patients could have strengthened the study. Furthermore, a control group on a lifestyle intervention program could help differentiate between weight loss effects and strictly procedural effects.

This study presents the long-term effects of a new transoral gastroplication treatment in morbidly obese patients. We show that this recently developed ACE stapler procedure was not only effective in reducing body weight as presented before [3], but also improved glycated haemoglobin levels and increased plasma adiponectin. Furthermore, whole transcriptome analysis suggested a marked downregulation of inflammatory gene sets in both the fundus and duodenum, coinciding with changes in plasma cytokines. Moreover, gene expression of ghrelin and its activating enzyme GOAT were reduced after gastroplication. The apparent reduction of inflammatory tone in the upper GI tract may be a consequence of an improved metabolic health status as associated with weight loss, or alternatively caused by the procedure itself.

In conclusion, this new transoral gastroplication treatment which induced significant weight loss and improved plasma levels of adiponectin and glycated haemoglobin coincides with a reduced inflammatory tone in the upper GI tract. The clinical relevance of our findings remains to be established, as there is still limited knowledge on the role of inflammatory pathways in the upper GI tract in obesity.

## Electronic supplementary material

ESM 1 (DOCX 33 kb)
