# Supplementary material for: Effect of Endoscopic Gastroplication on the Genome-Wide Transcriptome in the Upper Gastrointestinal Tract
Source: Obes Surg. 2016 Sep 13;27(3):740–8. doi: 10.1007/s11695-016-2356-0 (PMC5306242; doi:10.1007/s11695-016-2356-0)
Supplement: Supplementary file 1 — (DOCX 33 kb) [file 11695_2016_2356_MOESM1_ESM.docx]

Table S1: Full GSEA list of fundus, antrum and duodenum. Gene sets within several processes using conservative filtering (p<0.001 and FDR q<0.05) with their normalised enrichment scores (NES).

| **Fundus** |  |  |
| --- | --- | --- |
| **Process** | **Gene set** | **NES** |
| Antigen presentation and processing | INTERFERON.SIGNALING | -1.82327 |
|  | KEGG_ALLOGRAFT.REJECTION | -2.51834 |
|  | KEGG_VIRAL.MYOCARDITIS | -2.4598 |
|  | WP2739.AMYLOIDS | -2.07757 |
|  | KEGG_TUBERCULOSIS | -2.35037 |
|  | KEGG_TYPE.I.DIABETES.MELLITUS | -2.6364 |
|  | KEGG_TOXOPLASMOSIS | -1.86798 |
|  | INTERFERON.GAMMA.SIGNALING | -2.2216 |
|  | KEGG_SYSTEMIC.LUPUS.ERYTHEMATOSUS | -2.86087 |
|  | KEGG_ASTHMA | -2.34147 |
|  | CYTOKINE.SIGNALING.IN.IMMUNE.SYSTEM | -1.76089 |
|  | KEGG_AUTOIMMUNE.THYROID.DISEASE | -2.49038 |
|  | KEGG_STAPHYLOCOCCUS.AUREUS.INFECTION | -2.82522 |
|  | WP1835.INTERFERON.ALPHA.BETA.SIGNALING | -1.83516 |
|  | KEGG_CHAGAS.DISEASE.AMERICAN.TRYPANOSOMIASIS. | -2.24942 |
|  | KEGG_INTESTINAL.IMMUNE.NETWORK.FOR.IGA.PRODUCTION | -2.33433 |
|  | KEGG_CELL.ADHESION.MOLECULES.CAMS. | -2.59037 |
|  | WP1836.INTERFERON.GAMMA.SIGNALING | -2.19636 |
|  | KEGG_GRAFT.VERSUS.HOST.DISEASE | -2.67827 |
|  | KEGG_ANTIGEN.PROCESSING.AND.PRESENTATION | -2.57427 |
|  | WP2328.ALLOGRAFT.REJECTION | -2.33522 |
|  | KEGG_LEISHMANIASIS | -2.43961 |
|  | KEGG_INFLUENZA.A | -1.70275 |
|  | KEGG_RHEUMATOID.ARTHRITIS | -1.97922 |
| Complement system | WP558.COMPLEMENT.AND.COAGULATION.CASCADES | -2.64551 |
|  | WP2806.HUMAN.COMPLEMENT.SYSTEM | -2.14534 |
|  | WP2775.TOLL.LIKE.RECEPTORS.CASCADES | -2.24403 |
|  | WP1829.IMMUNOREGULATORY.INTERACTIONS.BETWEEN.A.LYMPHOID.AND.A.NON.LYMPHOID.CELL | -2.75048 |
|  | WP1798.COMPLEMENT.CASCADE | -2.59545 |
|  | WP1794.CELL.SURFACE.INTERACTIONS.AT.THE.VASCULAR.WALL | -2.22474 |
|  | SCAVENGING.OF.HEME.FROM.PLASMA | -1.98708 |
|  | RESPONSE.TO.ELEVATED.PLATELET.CYTOSOLIC.CA2. | -2.11246 |
|  | PLATELET.DEGRANULATION | -2.13783 |
|  | PLATELET.ACTIVATION.SIGNALING.AND.AGGREGATION | -1.87206 |
|  | NITRIC.OXIDE.STIMULATES.GUANYLATE.CYCLASE | -2.28073 |
|  | KEGG_COMPLEMENT.AND.COAGULATION.CASCADES | -2.67419 |
|  | INNATE.IMMUNE.SYSTEM | -1.92568 |
|  | INITIAL.TRIGGERING.OF.COMPLEMENT | -2.53372 |
|  | IMMUNOREGULATORY.INTERACTIONS.BETWEEN.A.LYMPHOID.AND.A.NON.LYMPHOID.CELL | -2.73543 |
|  | HEMOSTASIS | -2.03207 |
|  | FORMATION.OF.FIBRIN.CLOT.CLOTTING.CASCADE. | -1.94238 |
|  | FCGR.ACTIVATION | -2.31351 |
|  | FCERI.MEDIATED.CA.2.MOBILIZATION | -2.07759 |
|  | CREATION.OF.C4.AND.C2.ACTIVATORS | -2.33675 |
|  | COMPLEMENT.CASCADE | -2.64418 |
|  | CGMP.EFFECTS | -2.14792 |
|  | CELL.SURFACE.INTERACTIONS.AT.THE.VASCULAR.WALL | -2.05582 |
| T-cell receptor signaling | PD.1.SIGNALING | -2.40159 |
|  | TCR.SIGNALING | -2.3198 |
|  | DOWNSTREAM.TCR.SIGNALING | -2.18912 |
|  | COSTIMULATION.BY.THE.CD28.FAMILY | -2.0165 |
|  | WP1927.TCR.SIGNALING | -2.21863 |
|  | PHOSPHORYLATION.OF.CD3.AND.TCR.ZETA.CHAINS | -2.30236 |
|  | GENERATION.OF.SECOND.MESSENGER.MOLECULES | -2.67015 |
|  | WP1799.COSTIMULATION.BY.THE.CD28.FAMILY | -2.11037 |
| Metabolism of xenobiotics | KEGG_CHEMICAL.CARCINOGENESIS | -2.10708 |
|  | GLUTATHIONE.CONJUGATION | -1.77924 |
|  | KEGG_METABOLISM.OF.XENOBIOTICS.BY.CYTOCHROME.P450 | -2.23614 |
|  | KEGG_DRUG.METABOLISM.CYTOCHROME.P450 | -2.02946 |
|  | KEGG_DILATED.CARDIOMYOPATHY | -1.97885 |
| Cardiomyopathy | WP2118.ARRHYTHMOGENIC.RIGHT.VENTRICULAR.CARDIOMYOPATHY | -1.96139 |
|  | KEGG_ARRHYTHMOGENIC.RIGHT.VENTRICULAR.CARDIOMYOPATHY.ARVC. | -1.99686 |
|  | KEGG_HYPERTROPHIC.CARDIOMYOPATHY.HCM. | -1.95811 |
| Muscle contraction | MUSCLE.CONTRACTION | -2.04807 |
|  | WP1864.MUSCLE.CONTRACTION | -2.02105 |
|  | SMOOTH.MUSCLE.CONTRACTION | -2.15383 |
| Receptors | PEPTIDE.LIGAND.BINDING.RECEPTORS | -1.73849 |
|  | WP24.PEPTIDE.GPCRS | -1.82179 |
|  | CHEMOKINE.RECEPTORS.BIND.CHEMOKINES | -2.16619 |
| Other | WP619.TYPE.II.INTERFERON.SIGNALING.IFNG. | -2.14764 |
|  | BIOC_NKTPATHWAY | -2.18916 |
|  | MHC.CLASS.II.ANTIGEN.PRESENTATION | -1.70662 |
|  | AMYLOIDS | -1.81128 |
|  | KEGG_HEMATOPOIETIC.CELL.LINEAGE | -1.92881 |
|  | KEGG_LYSOSOME | -1.73537 |
|  | WP560.TGF.BETA.SIGNALING.PATHWAY | -1.75296 |
|  | KEGG_MALARIA | -1.94819 |
|  | KEGG_LONG.TERM.DEPRESSION | -1.73225 |
|  | MOLECULES.ASSOCIATED.WITH.ELASTIC.FIBRES | -2.00971 |
|  | KEGG_PERTUSSIS | -2.42017 |
|  | WP1833.INTEGRIN.CELL.SURFACE.INTERACTIONS | -1.98508 |
|  | ARACHIDONIC.ACID.METABOLISM | -1.95268 |
|  | KEGG_LEUKOCYTE.TRANSENDOTHELIAL.MIGRATION | -1.8182 |
|  | G.PROTEIN.MEDIATED.EVENTS | -1.70815 |
|  | WP2431.SPINAL.CORD.INJURY | -2.15311 |

| **Antrum** |  |  |
| --- | --- | --- |
| **Process** | **Gene set** | **NES** |
| Cell cycle | KEGG_ALLOGRAFT.REJECTION | -1.85431 |
|  | FORMATION.OF.THE.BETA.CATENIN.TCF.TRANSACTIVATING.COMPLEX | 1.970776 |
|  | WP1775.CELL.CYCLE.CHECKPOINTS | 2.175551 |
|  | RNA.POLYMERASE.I.PROMOTER.OPENING | 2.196208 |
|  | CELL.CYCLE | 2.353103 |
|  | TRANSCRIPTIONAL.REGULATION.BY.SMALL.RNAS | 1.887323 |
|  | MITOTIC.METAPHASE.AND.ANAPHASE | 2.200172 |
|  | ACTIVATION.OF.APC.C.AND.APC.C.CDC20.MEDIATED.DEGRADATION.OF.MITOTIC.PROTEINS | 1.747528 |
|  | NORC.NEGATIVELY.REGULATES.RRNA.EXPRESSION | 1.875334 |
|  | WP179.CELL.CYCLE | 2.256023 |
|  | ORC1.REMOVAL.FROM.CHROMATIN | 1.964168 |
|  | REGULATION.OF.DNA.REPLICATION | 2.05806 |
|  | REGULATION.OF.APC.C.ACTIVATORS.BETWEEN.G1.S.AND.EARLY.ANAPHASE | 1.695511 |
|  | CDC20.PHOSPHO.APC.C.MEDIATED.DEGRADATION.OF.CYCLIN.A | 1.642509 |
|  | CYCLIN.A.CDK2.ASSOCIATED.EVENTS.AT.S.PHASE.ENTRY | 1.863858 |
|  | ACTIVATION.OF.ATR.IN.RESPONSE.TO.REPLICATION.STRESS | 2.31258 |
|  | WP1874.NUCLEOSOME.ASSEMBLY | 2.30575 |
|  | WP1858.MITOTIC.G1.G1.S.PHASES | 2.262116 |
|  | CYCLIN.E.ASSOCIATED.EVENTS.DURING.G1.S.TRANSITION | 1.828069 |
|  | WP2369.HISTONE.MODIFICATIONS | 2.202452 |
|  | WP2739.AMYLOIDS | 2.570812 |
|  | TCF.DEPENDENT.SIGNALING.IN.RESPONSE.TO.WNT | 1.680383 |
|  | REMOVAL.OF.LICENSING.FACTORS.FROM.ORIGINS | 1.97605 |
|  | WP1928.TELOMERE.MAINTENANCE | 2.337343 |
|  | KEGG_DNA.REPLICATION | 2.309638 |
|  | CONDENSATION.OF.PROPHASE.CHROMOSOMES | 2.472027 |
|  | PRC2.METHYLATES.HISTONES.AND.DNA | 2.179259 |
|  | MITOTIC.ANAPHASE | 2.227793 |
|  | SEPARATION.OF.SISTER.CHROMATIDS | 2.265669 |
|  | WP45.G1.TO.S.CELL.CYCLE.CONTROL | 2.216835 |
|  | MEIOTIC.RECOMBINATION | 1.907697 |
|  | WP2757.MITOTIC.METAPHASE.AND.ANAPHASE | 2.385166 |
|  | MITOTIC.G1.G1.S.PHASES | 2.388205 |
|  | SIGNALING.BY.WNT | 1.675156 |
|  | WP2698.MEIOTIC.RECOMBINATION | 1.839597 |
|  | WP1898.REGULATION.OF.DNA.REPLICATION | 1.942631 |
|  | NUCLEOSOME.ASSEMBLY | 2.48161 |
|  | G0.AND.EARLY.G1 | 2.180428 |
|  | WP2652.MITOTIC.PROMETAPHASE | 2.602052 |
|  | SYNTHESIS.OF.DNA | 2.247251 |
|  | MISSPLICED.LRP5.MUTANTS.HAVE.ENHANCED.BETA.CATENIN.DEPENDENT.SIGNALING | 1.682867 |
|  | WP2446.RB.IN.CANCER | 2.758937 |
|  | MEIOSIS | 1.730538 |
|  | DNA.METHYLATION | 2.263346 |
|  | MITOTIC.PROMETAPHASE | 2.418687 |
|  | M.PHASE | 2.290548 |
|  | NEGATIVE.EPIGENETIC.REGULATION.OF.RRNA.EXPRESSION | 1.848622 |
|  | MITOTIC.G2.G2.M.PHASES | 1.640014 |
|  | ACTIVATION.OF.THE.PRE.REPLICATIVE.COMPLEX | 2.57844 |
|  | S.PHASE | 2.294708 |
|  | RNA.POLYMERASE.I.PROMOTER.CLEARANCE | 1.710418 |
|  | DNA.REPLICATION | 2.317099 |
|  | KEGG_SYSTEMIC.LUPUS.ERYTHEMATOSUS | 2.425107 |
|  | XAV939.INHIBITS.TANKYRASE.STABILIZING.AXIN | 1.679086 |
|  | TELOMERE.MAINTENANCE | 2.313958 |
|  | CELL.CYCLE.CHECKPOINTS | 2.155747 |
|  | MITOTIC.M.M.G1.PHASES | 2.536521 |
|  | G1.S.SPECIFIC.TRANSCRIPTION | 2.569735 |
|  | G2.M.CHECKPOINTS | 2.369734 |
|  | WP1925.SYNTHESIS.OF.DNA | 2.370287 |
|  | CELLULAR.SENESCENCE | 1.838437 |
|  | WP2785.M.G1.TRANSITION | 2.181483 |
|  | KEGG_CELL.CYCLE | 2.147555 |
|  | WP2361.GASTRIC.CANCER.NETWORK.1 | 2.383789 |
|  | PACKAGING.OF.TELOMERE.ENDS | 2.018427 |
|  | CELL.CYCLE.MITOTIC | 2.420983 |
|  | DEPOSITION.OF.NEW.CENPA.CONTAINING.NUCLEOSOMES.AT.THE.CENTROMERE | 2.464646 |
|  | WP466.DNA.REPLICATION | 2.526486 |
|  | CHROMOSOME.MAINTENANCE | 2.485708 |
|  | RNF.MUTANTS.SHOW.ENHANCED.WNT.SIGNALING.AND.PROLIFERATION | 1.690068 |
|  | REGULATORY.RNA.PATHWAYS | 1.751141 |
|  | POLO.LIKE.KINASE.MEDIATED.EVENTS | 1.939158 |
|  | SENESCENCE.ASSOCIATED.SECRETORY.PHENOTYPE.SASP. | 1.861184 |
|  | APOPTOSIS | 1.650792 |
|  | M.G1.TRANSITION | 2.204365 |
|  | ASSEMBLY.OF.THE.PRE.REPLICATIVE.COMPLEX | 2.020908 |
|  | E2F.MEDIATED.REGULATION.OF.DNA.REPLICATION | 2.469653 |
|  | DNA.STRAND.ELONGATION | 2.291611 |
|  | KEGG_ALCOHOLISM | 2.205282 |
|  | EPIGENETIC.REGULATION.OF.GENE.EXPRESSION | 1.87112 |
|  | EXTENSION.OF.TELOMERES | 2.140079 |
|  | G1.S.TRANSITION | 2.515779 |
|  | WP2772.S.PHASE | 2.367719 |
|  | RESOLUTION.OF.SISTER.CHROMATID.COHESION | 2.435047 |
|  | DNA.REPLICATION.PRE.INITIATION | 2.262319 |
| Transcription and translation | WP2710.NONSENSE.MEDIATED.DECAY | 1.956784 |
|  | WP1889.PROCESSING.OF.CAPPED.INTRON.CONTAINING.PRE.MRNA | 1.987702 |
|  | WP411.MRNA.PROCESSING | 2.125963 |
|  | MRNA.SPLICING | 1.973784 |
|  | WP1811.EUKARYOTIC.TRANSLATION.ELONGATION | 2.037809 |
|  | KEGG_SPLICEOSOME | 1.874981 |
|  | WP1812.EUKARYOTIC.TRANSLATION.INITIATION | 1.890107 |
|  | WP2737.SRP.DEPENDENT.COTRANSLATIONAL.PROTEIN.TARGETING.TO.MEMBRANE | 1.809973 |
|  | WP477.CYTOPLASMIC.RIBOSOMAL.PROTEINS | 2.03664 |
|  | WP2683.INFLUENZA.LIFE.CYCLE | 2.231637 |
|  | PROCESSING.OF.CAPPED.INTRON.CONTAINING.PRE.MRNA | 1.966031 |
|  | MRNA.SPLICING.MAJOR.PATHWAY | 1.968078 |
|  | WP1813.EUKARYOTIC.TRANSLATION.TERMINATION | 1.982535 |
| Extracellular matrix | EXTRACELLULAR.MATRIX.ORGANIZATION | 2.066144 |
|  | WP2725.COLLAGEN.BIOSYNTHESIS.AND.MODIFYING.ENZYMES | 1.933647 |
|  | KEGG_FOCAL.ADHESION | 1.739328 |
|  | KEGG_ECM.RECEPTOR.INTERACTION | 2.178188 |
|  | DEGRADATION.OF.THE.EXTRACELLULAR.MATRIX | 1.925916 |
|  | WP306.FOCAL.ADHESION | 1.776627 |
|  | COLLAGEN.BIOSYNTHESIS.AND.MODIFYING.ENZYMES | 2.084609 |
|  | WP185.INTEGRIN.MEDIATED.CELL.ADHESION | 1.66432 |
|  | INTEGRIN.CELL.SURFACE.INTERACTIONS | 1.809606 |
|  | COLLAGEN.FORMATION | 2.233561 |
|  | ELASTIC.FIBRE.FORMATION | 1.798224 |
|  | WP2798.ASSEMBLY.OF.COLLAGEN.FIBRILS.AND.OTHER.MULTIMERIC.STRUCTURES | 2.186452 |
|  | ASSEMBLY.OF.COLLAGEN.FIBRILS.AND.OTHER.MULTIMERIC.STRUCTURES | 2.156891 |
| Cellular metabolism | REGULATION.OF.PYRUVATE.DEHYDROGENASE.PDH.COMPLEX | -1.96206 |
|  | WP1902.RESPIRATORY.ELECTRON.TRANSPORT.ATP.SYNTHESIS.BY.CHEMIOSMOTIC.COUPLING.AND.HEAT.PRODUCTION.BY.UNCOUPLING.PROTEINS. | -2.17445 |
|  | WP111.ELECTRON.TRANSPORT.CHAIN | -2.04609 |
|  | KEGG_COLLECTING.DUCT.ACID.SECRETION | -1.96777 |
|  | KEGG_OXIDATIVE.PHOSPHORYLATION | -2.24102 |
|  | THE.CITRIC.ACID.TCA.CYCLE.AND.RESPIRATORY.ELECTRON.TRANSPORT | -2.43706 |
|  | PYRUVATE.METABOLISM.AND.CITRIC.ACID.TCA.CYCLE | -2.15426 |
|  | RESPIRATORY.ELECTRON.TRANSPORT | -2.26064 |
|  | WP623.OXIDATIVE.PHOSPHORYLATION | -2.21162 |
|  | WP2766.THE.CITRIC.ACID.TCA.CYCLE.AND.RESPIRATORY.ELECTRON.TRANSPORT. | -2.00618 |
|  | RESPIRATORY.ELECTRON.TRANSPORT.ATP.SYNTHESIS.BY.CHEMIOSMOTIC.COUPLING.AND.HEAT.PRODUCTION.BY.UNCOUPLING.PROTEINS. | -2.279 |
| Cell junctions | CELL.CELL.JUNCTION.ORGANIZATION | 1.957272 |
|  | CELL.JUNCTION.ORGANIZATION | 2.081496 |
|  | WP1793.CELL.JUNCTION.ORGANIZATION | 1.780006 |
|  | CELL.CELL.COMMUNICATION | 1.77621 |
|  | TIGHT.JUNCTION.INTERACTIONS | 2.166633 |
| Protein folding | CHAPERONIN.MEDIATED.PROTEIN.FOLDING | 1.957393 |
|  | WP1892.PROTEIN.FOLDING | 2.131613 |
|  | FORMATION.OF.TUBULIN.FOLDING.INTERMEDIATES.BY.CCT.TRIC | 2.236554 |
|  | PREFOLDIN.MEDIATED.TRANSFER.OF.SUBSTRATE.TO.CCT.TRIC | 2.431509 |
|  | COOPERATION.OF.PREFOLDIN.AND.TRIC.CCT.IN.ACTIN.AND.TUBULIN.FOLDING | 2.363184 |
| Cardiomyopathy | KEGG_ARRHYTHMOGENIC.RIGHT.VENTRICULAR.CARDIOMYOPATHY.ARVC. | 2.307521 |
|  | KEGG_HYPERTROPHIC.CARDIOMYOPATHY.HCM. | 1.86531 |
|  | KEGG_DILATED.CARDIOMYOPATHY | 2.017088 |
|  | WP2118.ARRHYTHMOGENIC.RIGHT.VENTRICULAR.CARDIOMYOPATHY | 2.31039 |
| Kinesins (move along microtubule filaments) | KINESINS | 2.299073 |
|  | WP1842.KINESINS | 2.420609 |
|  | MHC.CLASS.II.ANTIGEN.PRESENTATION | 1.797628 |
| Muscle contraction | WP1864.MUSCLE.CONTRACTION | 2.033824 |
|  | MUSCLE.CONTRACTION | 2.058598 |
|  | SMOOTH.MUSCLE.CONTRACTION | 2.151354 |
| Other | WP1422.SPHINGOLIPID.METABOLISM | -2.01252 |
|  | WP2377.INTEGRATED.PANCREATIC.CANCER.PATHWAY | 1.697525 |
|  | KEGG_SALMONELLA.INFECTION | 1.824445 |
|  | KEGG_PROPANOATE.METABOLISM | -1.91319 |
|  | GAP.JUNCTION.TRAFFICKING | 1.829106 |
|  | AXON.GUIDANCE | 1.769707 |
|  | WP143.FATTY.ACID.BETA.OXIDATION | -2.18356 |
|  | KEGG_VALINE.LEUCINE.AND.ISOLEUCINE.DEGRADATION | -2.03598 |
|  | BRANCHED.CHAIN.AMINO.ACID.CATABOLISM | -2.29066 |
|  | KEGG_REGULATION.OF.AUTOPHAGY | -1.98463 |
|  | WP1831.INTEGRATION.OF.ENERGY.METABOLISM | 1.920028 |
|  | WP2664.GASTRIN.CREB.SIGNALLING.PATHWAY.VIA.PKC.AND.MAPK | 1.687694 |
|  | WP368.MITOCHONDRIAL.LC.FATTY.ACID.BETA.OXIDATION | -1.97753 |
|  | WP536.CALCIUM.REGULATION.IN.THE.CARDIAC.CELL | 1.945329 |
|  | GLUTATHIONE.CONJUGATION | -1.8823 |
|  | INTEGRATION.OF.ENERGY.METABOLISM | 1.788005 |
|  | GENERATION.OF.SECOND.MESSENGER.MOLECULES | -1.8954 |
|  | EPH.EPHRIN.SIGNALING | 1.729218 |
|  | GENERIC.TRANSCRIPTION.PATHWAY | -2.103 |

| Duodenum |  |  |
| --- | --- | --- |
| Process | Gene set | NES |
| Lipid metabolism | PHOSPHOLIPID.METABOLISM | 1.83 |
|  | KEGG_FAT.DIGESTION.AND.ABSORPTION | 2.42 |
|  | FATTY.ACID.TRIACYLGLYCEROL.AND.KETONE.BODY.METABOLISM | 1.77 |
|  | REGULATION.OF.CHOLESTEROL.BIOSYNTHESIS.BY.SREBP.SREBF. | 2.14 |
|  | WP197.CHOLESTEROL.BIOSYNTHESIS | 2.20 |
|  | PEROXISOMAL.LIPID.METABOLISM | 1.99 |
|  | ACTIVATION.OF.GENE.EXPRESSION.BY.SREBF.SREBP. | 2.11 |
|  | KEGG_PEROXISOME | 1.93 |
|  | SPHINGOLIPID.DE.NOVO.BIOSYNTHESIS | 1.81 |
|  | KEGG_GLYCEROPHOSPHOLIPID.METABOLISM | 2.18 |
|  | WP2740.GLYCEROPHOSPHOLIPID.BIOSYNTHESIS | 1.98 |
|  | CHOLESTEROL.BIOSYNTHESIS | 2.07 |
|  | METABOLISM.OF.LIPIDS.AND.LIPOPROTEINS | 2.07 |
|  | GLYCEROPHOSPHOLIPID.BIOSYNTHESIS | 1.92 |
|  | KEGG_SPHINGOLIPID.METABOLISM | 2.00 |
|  | WP1817.FATTY.ACID.TRIACYLGLYCEROL.AND.KETONE.BODY.METABOLISM | 1.81 |
| Metabolism of xenobiotics | WP702.METAPATHWAY.BIOTRANSFORMATION | 2.09 |
|  | KEGG_CHEMICAL.CARCINOGENESIS | 2.31 |
|  | KEGG_METABOLISM.OF.XENOBIOTICS.BY.CYTOCHROME.P450 | 2.19 |
|  | WP43.OXIDATION.BY.CYTOCHROME.P450 | 2.03 |
|  | KEGG_DRUG.METABOLISM.CYTOCHROME.P450 | 1.93 |
|  | CYTOCHROME.P450.ARRANGED.BY.SUBSTRATE.TYPE | 2.07 |
|  | BIOLOGICAL.OXIDATIONS | 2.23 |
|  | PHASE.1.FUNCTIONALIZATION.OF.COMPOUNDS | 2.11 |
|  | PHASE.II.CONJUGATION | 1.73 |
|  | KEGG_RETINOL.METABOLISM | 2.09 |
| Chaperones (protein) | WP2667.ACTIVATION.OF.CHAPERONE.GENES.BY.XBP1.S. | -1.97 |
|  | UNFOLDED.PROTEIN.RESPONSE.UPR. | -2.33 |
|  | XBP1.S.ACTIVATES.CHAPERONE.GENES | -2.00 |
|  | IRE1ALPHA.ACTIVATES.CHAPERONES | -2.10 |
| Respiratory electron transport chain | WP1902.RESPIRATORY.ELECTRON.TRANSPORT.ATP.SYNTHESIS.BY.CHEMIOSMOTIC.COUPLING.AND.HEAT.PRODUCTION.BY.UNCOUPLING.PROTEINS. | 1.97 |
|  | RESPIRATORY.ELECTRON.TRANSPORT | 1.91 |
|  | WP111.ELECTRON.TRANSPORT.CHAIN | 1.93 |
|  | RESPIRATORY.ELECTRON.TRANSPORT.ATP.SYNTHESIS.BY.CHEMIOSMOTIC.COUPLING.AND.HEAT.PRODUCTION.BY.UNCOUPLING.PROTEINS. | 1.86 |
| Innate immunity | INITIAL.TRIGGERING.OF.COMPLEMENT | -1.91 |
|  | IMMUNOREGULATORY.INTERACTIONS.BETWEEN.A.LYMPHOID.AND.A.NON.LYMPHOID.CELL | -1.93 |
|  | FCGR.ACTIVATION | -1.95 |
| Cell cycle | WP1928.TELOMERE.MAINTENANCE | 2.18 |
|  | WP466.DNA.REPLICATION | 1.93 |
|  | KEGG_DNA.REPLICATION | 2.05 |
|  | TELOMERE.MAINTENANCE | 1.89 |
|  | WP1925.SYNTHESIS.OF.DNA | 1.72 |
|  | DNA.METHYLATION | 1.96 |
|  | WP2739.AMYLOIDS | 1.98 |
| Other | PLATELET.DEGRANULATION | -1.94 |
|  | KEGG_BILE.SECRETION | 2.10 |
|  | BIOSYNTHESIS.OF.THE.N.GLYCAN.PRECURSOR.DOLICHOL.LIPID.LINKED.OLIGOSACCHARIDE.LLO.AND.TRANSFER.TO.A.NASCENT.PROTEIN | -1.99 |
|  | PPARA_TARGETS | 2.26 |
|  | KEGG_PROTEIN.EXPORT | -2.04 |
|  | KEGG_PROTEIN.PROCESSING.IN.ENDOPLASMIC.RETICULUM | -2.26 |
|  | KEGG_DRUG.METABOLISM.OTHER.ENZYMES | 1.87 |
|  | KEGG_PPAR.SIGNALING.PATHWAY | 2.05 |
|  | APOPTOTIC.EXECUTION.PHASE | 1.83 |
|  | KEGG_GLYCEROLIPID.METABOLISM | 1.93 |
|  | WP2806.HUMAN.COMPLEMENT.SYSTEM | -2.01 |
|  | WP716.VITAMIN.A.AND.CAROTENOID.METABOLISM | 2.17 |
|  | KEGG_STAPHYLOCOCCUS.AUREUS.INFECTION | -2.30 |
|  | KEGG_INTESTINAL.IMMUNE.NETWORK.FOR.IGA.PRODUCTION | -1.90 |
|  | WP2840.HAIR.FOLLICLE.DEVELOPMENT.CYTODIFFERENTIATION.PART.3.OF.3. | -1.96 |
|  | KEGG_PROTEIN.DIGESTION.AND.ABSORPTION | 1.92 |
|  | KEGG_HOMOLOGOUS.RECOMBINATION | 2.05 |
|  | WP2431.SPINAL.CORD.INJURY | -1.97 |
|  | KEGG_FATTY.ACID.DEGRADATION | 1.99 |
